# Sponsorship in non-commercial clinical trials: definitions, challenges and the role of Good Clinical Practices guidelines

**DOI:** 10.1186/s12914-015-0073-8

**Published:** 2015-12-30

**Authors:** Raffaella Ravinetto, Katelijne De Nys, Marleen Boelaert, Ermias Diro, Graeme Meintjes, Yeka Adoke, Harry Tagbor, Minne Casteels

**Affiliations:** Clinical Sciences Department, Institute of Tropical Medicine Antwerp, Antwerp, Belgium; Clinical Pharmacology and Pharmacotherapy, KU Leuven, Leuven, Belgium; Clinical Trial Center, University Hospitals Leuven, Leuven, Belgium; Public Health Department, Institute of Tropical Medicine Antwerp, Antwerp, Belgium; Department of Internal Medicine, University of Gondar, Gondar, Ethiopia; Institute of Infectious Disease and Molecular Medicine and Department of Medicine, University of Cape Town, Cape Town, South Africa; College of Health Sciences, Makerere University School of Public Health, Kampala, Uganda; School of Public Health, Kwame Nkrumah University of Science and Technology, Kumasi, Ghana

## Abstract

**Background:**

Non-commercial clinical research plays an increasingly essential role for global health. Multiple partners join in international consortia that operate under the limited timeframe of a specific funding period. One organisation (the sponsor) designs and carries out the trial in collaboration with research partners, and is ultimately responsible for the trial’s scientific, ethical, regulatory and legal aspects, while another organization, generally in the North (the funder), provides the external funding and sets funding conditions. Even if external funding mechanisms are key for most non-commercial research, the dependence on an external funder’s policies may heavily influence the choices of a sponsor. In addition, the competition for accessing the available external funds is great, and non-commercial sponsors may not be in a position to discuss or refuse standard conditions set by a funder.

To see whether the current definitions adequately address the intricacies of sponsorship in externally-funded trials, we looked at how a “sponsor” of clinical trials is defined in selected international guidelines, with particular focus on international Good Clinical Practices codes, and in selected European and African regulations/legislations.

**Discussion:**

Our limited analysis suggests that the sponsors definition from the 1995 WHO Good Clinical Practices code has been integrated as such into many legislations, guidelines and regulations, and that it is not adequate to cover today’s reality of funding arrangements in global health, where the legal responsibility and the funding source are de facto split. In agreement with other groups, we suggest that the international Good Clinical Practices codes should be updated to reflect the reality of non-commercial clinical research. In particular, they should explicitly include the distinction between commercial and non-commercial sponsors, and provide guidance to non-commercial sponsors for negotiating with external funding agencies and other research counterparts.

**Summary:**

Non-commercial sponsors of clinical trials should surely invest in the development of adequate legal, administrative and management skills. By acknowledging their role and specificities, and by providing them with adapted guidance, the international Good Clinical Practices codes would provide valuable guidance and support to non-commercial clinical research, whose relevance for global health is increasingly evident.

## Background

The North–south divide in access to health is very large. Clinical research & development (R&D) follows a similar pattern, and this despite rapid evolution in the field of clinical trials in the South. There is a clear tendency to relocate trials to resource-poor settings [1, 2], either for reasons of *external validity*, i.e. to challenge findings obtained in the North on new drugs and devices in a variety of epidemiological settings and populations; or for *convenience* reasons, represented by lower costs, less stringent review, and potentially higher recruitment rates; or for *global health* reasons, when the choice for a location in the South is driven by the need to address the specific health needs of the local population.

In recent years, there has been a significant increase in clinical research carried out under non-commercial North–south collaborations, prompted by *global health reasons*. Non-commercial clinical research as well as public-private partnerships are essential for R&D of medical products of no direct commercial interest, for assessing the effectiveness and feasibility of medical products and health interventions in specific contexts/groups, and for providing independent evaluation of such products and interventions [3, 4]. The increased availability of funds and technological investment for global health [5, 6], by philanthropic charities, foundations, public-private partnerships, bilateral/multilateral aid, and sometimes pharmaceutical companies, provide opportunities for non-commercial research groups to design, carry out and sponsor scientifically sound clinical research in traditionally neglected areas, e.g. infectious neglected diseases and tuberculosis.

In commercial research, the same organization (usually, a pharmaceutical company) funds, designs and carries out a trial. But the new context of North–South collaborations often leads to complex arrangements, with multiple partners joining in international consortia that often have an ad hoc structure and operate under the limited timeframe of a specific funding period.

Only in few cases will academic sponsors be able to conduct trials without external funding. Generally, one agency in the North (usually a foundation or a public authority) provides the funding and sets conditions for such funding, and another organization (henceforward called the sponsor), usually but not always located in the North, designs and conducts the trial, in collaboration with research partners. The financial arrangements between the funding agency and the research partners are formalized in contracts defining respective roles and responsibilities, and they may be quite complex. The main contract is usually signed between the funder and the non-commercial research consortium, and it defines the conditions under which the agreed funding will be disbursed. The liability issues generated by the testing of new medicines, devices or protocols should be carefully considered in contractual agreements. The set-up of a complex multi-institutional partnership may sometimes be at odds with the concept of “single sponsorship“, which was developed for ensuring the protection of participants in the context of commercial trials. Single sponsorship is rooted in the need to clearly identify the legal responsibility, and does not hinge upon the “funding” aspect. Noteworthy, most funding agencies are unwillingly to take on the role of sponsor and often they may not be suitable for this.

Non-commercial international research is not immune from any risks for exploitation, depending on how it is designed and conducted and on how findings are implemented and disseminated. Exploitation can arise when the ambitions of the academic sponsors or the strategical plans of the funders prevail on the interests of the communities. Hence the importance to strengthen the regulatory framework for non-commercial research, starting with the key question on the legal responsibility.

The concept of sponsorship merits closer scrutiny in this context. To enlighten the debate we examined how a “sponsor” of clinical trials is defined in selected international or national guidelines and legislation, and discuss whether current definitions adequately address the intricacies of sponsorship in externally (i.e. “outside of the sponsor”)-funded trials.

## Discussion

Among international guidelines, we considered those that most often inspire national legislations: the Declaration of Helsinki, the International Ethical Guidelines for Biomedical Research Involving Human Subjects (CIOMS), and the Good Clinical Practices (GCP) code of the World Health Organization (WHO) and of the International Conference for Harmonization (ICH). However, the Helsinki Declaration [7] and the CIOMS Guidelines [8] do not give a specific definition of what a “sponsor” is nor of its duties, even if both mention “sponsors” among the actors of medical research, and the CIOMS Guidelines also talk of “externally sponsored research” in relation to trials carried out in a different host country. Therefore, the Helsinki Declaration and the CIOMS Guidelines were excluded from the comparative analysis.

The selection of the international regulations and national guidelines/legislations included in the analysis was guided by our experience in North–South collaborative trials that bring together African and European institutions. Thus, we focused on the European and African regulatory environment. Among international regulations, we considered the European Union (EU) Directive, which is the main reference for European Member States and funding agencies. Among national guidelines/legislation, we considered the UK Clinical Trials Regulation, which is the main reference for some Commonwealth countries and for UK funding agencies, and the regulations/legislation of host countries of our research projects, i.e. Belgium, South Africa, Ethiopia, Uganda and Ghana.

### Sponsor’s definitions

Table 1 gives an overview of the definition of sponsor in the selected documents. The WHO/GCP Guidelines, issued in 1995 [9], define the sponsor as “an individual, a company, an institution or an organization which takes responsibility for the initiation, management *and/or financing* of a clinical trial”. The ICH/GCP Guidelines, issued in 1996 [10], use exactly the same definition, which leaves some degree of ambiguity about whether the sponsor should be primarily responsible for the “initiation and management” of a trial, or for its “financing”, or for both. This definition reflects the situation of the ‘90s, when clinical trials were mainly conducted in Western contexts by commercial sponsors assuming both roles. Both GCP codes accept the notion of “sponsor-investigator”, which only refers to an individual, thus is not applicable to non-commercial sponsors in global clinical research.

**Table 1:** Overview of the definition of sponsor

Guideline/regulation	Year	Sponsor’s definition
WHO GCP	1995	An individual, a company, an institution or an organization which takes responsibility for the initiation, management and/or financing of a clinical trial.
ICH GCP	1996	An individual, a company, an institution or an organization which takes responsibility for the initiation, management and/or financing of a clinical trial.
EU Directive	2002	An individual, company, institution or organization which takes responsibility for the initiation, management and/or financing of a clinical trial
Belgian Law	2004	An individual, company, institution or organization which takes responsibility for the initiation, management and/or financing of a clinical trial
UK Regulation	2004	Takes responsibility for the initiation, management and financing (or arranging the financing) of that trial.
South Africa GCP	2006	An individual, company, institution, or organisation which takes responsibility for the initiation, management, and/or financing of a clinical trial
Uganda Guidelines	2007	The sponsor is responsible for providing all the necessary financial support for initiation and completion of the research project
Uganda Guidelines	2014	The sponsor as such is not defined
Ghana GCP	2013	An individual, company, institution or organization which takes responsibility for the initiation, management and/or financing of a trial. This excludes an individual company, institution or organization which has been requested to provide money for a trial and does not benefit in any way from the results of the trial
EU Regulation	2014	An individual, company, institution or organisation which takes responsibility for the initiation, the management and for setting up the financing of the clinical trial
Ethiopia GCP	Not dated	An individual, a company, an institution or an organization which takes responsibility for the initiation, management and/or financing of a clinical trial.

The same definition was found in the South African [11] and Ethiopian [12] GCP guidelines (where the “funder” is mentioned but not defined), and in the 2001/20/EC/EU Directive [13]. The definition of the EU Directive was incorporated into national legislations, such as the Belgian Law of 2004 [14]. Noteworthy, the Belgian Law distinguishes between commercial and non-commercial sponsors and is explicit about the fact that the same responsibilities hold for both, although it assures some rights (such as data ownership) for non-commercial sponsors.

However, the recent European Regulation on Clinical Trials on Medicinal Products for Human Use [15] introduced an important clarification in the definition of a sponsor: “an individual, company, institution or organisation which takes responsibility for the initiation, the management *and for setting up the financing* of the clinical trial”. This definition clarifies that the sponsor is equally scientifically, legally and financially responsible but, by replacing the wording “financing” with the wording “setting up the financing”, it acknowledges that the budget can either come from the sponsor itself or from sources external to the research group. The UK regulators had already incorporated this nuance in their definition of sponsor, which is the one taking responsibility for the initiation, management *and financing (or arranging the financing)* of a trial [16].

The shift in the European legislation is in line with the growing attention paid to non-commercial research. Previously there was a description of the features of non-commercial clinical trials (i.e. those conducted without the participation of the pharmaceutical industry) and of non-commercial sponsors (i.e. universities, hospitals, public scientific organisations, non-profit institutions, patient organisations or individual researchers) [17]. The new regulation is much more explicit in acknowledging the importance of clinical trials conducted by non-commercial sponsors, which often rely on external funding from funds or charities.

Also in Africa, we find definitions which depart from the WHO/GCP guidelines, to be better rooted in the current reality. The previous (2007) Uganda National Guidelines for Research Involving Humans as Research Participants stated that the sponsor is responsible for *providing all the necessary financial support* for initiation and completion of the research project, while the detailed description of the sponsor’s responsibilities covered the different aspects of trial initiation and management [18]. The recent new Guidelines, issued in July 2014 [19], do not include anymore a formal definition of sponsor; however, the sponsor is still held responsible for providing all the necessary for implementation of the trial, including post-research obligations, while the detailed description of its responsibilities still covers the different aspects of trial initiation and management. In the Ghana GCP code [20], a clarification was added to the WHO/GCP definition, to clarify that an individual, company, institution or organization that provides money for a trial without benefiting from its results, is excluded from the definition of sponsor. Thus, for externally funded trials, the sponsor’s legal responsibility remains with the organization that initiates and manages the trial.

### Sponsor versus funder

This analysis suggests that the WHO/GCP definition of sponsor has been integrated as such into many international and national legislations, guidelines and regulations, and is not entirely adequate to cover the reality of funding arrangements in global health today (even if the difference between commercial and non-commercial sponsors is sometimes acknowledged). Some guidelines (South Africa, Ethiopia) mention the “funder” in addition to the sponsor, and some others (UK, Ghana, EU) reflect the fact that two or more different entities may respectively initiate/conduct a trial, and (co-)finance it. The former entity is the legal sponsor, while the latter is the external funder. But some ambiguity remains between “sponsor” and “funding agency”. This was further confirmed by the unsatisfactorily results of a complementary literature search: the wording “sponsor” was often inaccurately referred to as the organization that funds a research, i.e. in a “lay” meaning (like for the “sponsor” of an event), rather than in the GCP meaning. This ambiguity may contribute to the poor awareness of some inexperienced sponsors, especially in the non-commercial sector, of the scope of their own responsibilities.

The legal sponsor is ultimately responsible for the scientific, ethical, regulatory and legal aspects of the trial, and also for financial aspects (i.e., if an external funder withdraws, the sponsor will be responsible to look for funds to complete the trial). It is therefore its primary role and responsibility to ensure that sufficient resources are planned for full compliance with ethical and GCP requirements. Sponsors’ poor awareness of such requirements, which are described more in details in Table 2, may lead to underestimation of the overall study budget. In the specific case of externally-funded research, this will lead them to requesting insufficient financial resources from the funding agency.

**Table 2:** Overview of sponsor’s specific responsibilities in the international GCP codes

WHO GCP 1995	ICH GCP 1996
5.1 Selection of the Investigator(s)	5.1 Quality Assurance and Quality Control
5.2 Delegation of responsibilities	5.2 Contract Research Organization
5.3 Compliance with the protocol and procedures	5.3 Medical Expertise
5.4 Product information	5.4 Trial Design
5.5 Safety information	5.5 Trial Management, Data Handling, and Record Keeping
5.6 Investigational product	5.6 Investigator Selection
5.7 Trial management and handling of data	5.7 Allocation of Responsibilities
5.8 Standard operating procedures	5.8 Compensation to Subjects and Investigators
5.9 Compensation for subjects and investigators	5.9 Financing
5.10 Monitoring	5.10 Notification/Submission to Regulatory Authority(ies)
5.11 Quality assurance	5.11 Confirmation of Review by IRB/IEC
5.12 Study reports	5.12 Information on Investigational Product(s) (IPs)
5.13 Handling of adverse events	5.13 Manufacturing, Packaging, Labelling, and Coding IP(s)
5.14 Termination of the trial	5.14 Supplying and Handling IP(s)
	5.15 Record Access
	5.16 Safety Information
	5.17 Adverse Drug Reaction Reporting
	5.18 Monitoring
	5.19 Audit
	5.20 Noncompliance
	5.21 Premature Termination or Suspension of a Trial
	5.22 Clinical Trial/Study Reports
	5.23 Multicenter Trials

Non-commercial sponsors often face budgetary problems because of the poor flexibility of the external funding [21, 22]. Some funding bodies will not accept a miscellaneous budget line for contingency/incidentals and are unwilling to review or supplement the budget once agreements have been finalized (which often happens before detailed protocols have been developed, and even before the clinical sites’ needs are thoroughly assessed). The lack of flexibility is problematic also in other situations [23], e.g. when recruitment is slower than anticipated because of reasons beyond control of investigators, or when exchange rate with the local currency fluctuates over time: for instance, depreciation will make it more difficult to buy equipments abroad, while appreciation will decrease the local value of foreign funds.

As mentioned above, the poor awareness of GCP requirements may lead sponsors to requesting insufficient financial resources from the funding agency. But in other cases the budget awarded by the funding agency is much lower than the initially requested one, and may be insufficient to meet all costs required for full GCP-compliance, e.g. adequate external monitoring or data management set-up, costs that are unfortunately often the first to be cut in such situation.

### Negotiations between non-commercial sponsors and funders

One could argue that it is the sponsor’s responsibility to ensure that adequate funding and funding’s conditions are negotiated for carrying out a trial according to appropriate standards, and to reject unsatisfactory conditions. Even though some sponsors underestimate the budget, in reality many cost items may be impossible to foresee in advance [22]. Awareness of GCP-requirements and good negotiation skills are both essential to ensure that the positions of the funding agency and the research consortium are sufficiently reflected in the final contract, but in practice, contracts are often based on the standard templates of the funding agency. Many non-commercial sponsors lack a legal department with enough human resources to conduct such negotiations. According to an analysis of the Council in Health Research for Development (COHRED), for instance, various research institutions in Africa and Asia have weak contracting capacity [24]. In addition, non-commercial sponsors often find themselves in a position of power unbalance *vis-à-vis* the funding agency. The competition for accessing the external funds available for health research is great, and most North- or South-based non-commercial sponsors may not be in a position to negotiate the rules set by the funding agencies. They have to balance the risk of a contract that does not reflect particular quality requirements (“contractual risk”) versus the risk of being unable to conduct research relevant for a given population (“ethical risk”).

### The way forward?

In externally-funded clinical research, the legal responsibility and the funding source are often de facto split. The liability risks of sponsors, as well as those of researchers and other research actors have been described [25]. However, to our knowledge the responsibilities of the funding agencies have not been described so far, even if in non-commercial research many choices may be positively or negatively influenced/determined by their policy.

Some guidelines are subject to periodical update, which allows taking new challenges into account. An example of this dynamic is given by the revision of the Helsinki Declaration, preceded by a public debate. Conversely, the WHO/ and ICH/GCP Guidelines, which orient most national legislators, were issued respectively in 1995 and 1996 and never updated. A revision is urgently needed, to better reflect the current reality of clinical trials including the perspective of non-commercial research [23, 26]. More in particular, we are not aware of any processes for the update of the WHO/GCP. Concerning the ICH/GCP, an “Integrated addendum” was published on 11th June 2015, with the objective to”modernize the ICH E6 Guideline by supplementing with additional recommendations which will facilitate broad and consistent international implementation of new methodologies”. This draft text, now transmitted to the National Regulatory Authorities of the ICH region for internal and external consultation (accessed on 27th August 2015 at http://www.ich.org/products/guidelines/efficacy/efficacy-single/article/addendum-good-clinical-practice.html), does not address the definition of sponsor.

We suggest in the first place that the international GCP codes should include the distinction between commercial and non-commercial sponsors.

Non-commercial sponsors should pay special attention to the research legal framework and try to improve the contractual agreements with the funding agency, so that responsibilities and liability issues are fairly shared, and other important aspects are duly clarified. To be able to do so, non-commercial organisations that wish to act as sponsor in clinical trials should invest in the development of adequate legal, administrative and management skills, just as they do for scientific skills. This would also enable them to negotiate fair and meaningful contracts for other key-activities in clinical research, such as the supply of investigational products, the transfer and sharing of trials’ data and samples, and the policy insurance contract(s) [24]. To help cope with this, we suggest that the WHO/ and ICH/GCP Guidelines include as annexes some adapted model contract templates or standard checklists, with clauses for “reasonable flexibility”, to guide the negotiation with external funding agencies. The same applies to templates and guidance for negotiation with other research counterparts, e.g. insurance policies, data and material transfer agreements etc.

### Limitations of this analysis

Our analysis is meant to launch a debate aiming at better legislation on non-commercial sponsorships, based on the analysis of a sample of guidelines, laws and regulations. The international regulations and national guidelines/legislations included in this analysis were selected with focus on the European and (English-speaking) African regulatory environment. This led to exclusion of other influential guidelines and legislations, such as the ones from the United States, India, and Brazil, so more research is needed to investigate this issue in more regulatory environments.

While focusing on the sponsor’s definition and role, we did not look at the complex dynamics that may exist within North–South research consortia, or at the possible power unbalance between Northern and Southern partners. As noted by Hoekman and colleagues, despite the increasing globalization of clinical trials, the scientific leadership of research tends to remain rooted in the North [27], which may be translated into unfair benefit sharing with local researchers, institutions and communities in the South. This phenomenon should be investigated more in-depth in its own right.

## Conclusion

The current definitions of “sponsor” in clinical research do not reflect the challenges met by non-commercial sponsors in externally-funded research, especially but not only in the South, and in particular they do not adequately cover the reality of funding arrangements in clinical research in a global health context today. A revision of the WHO/ and ICH/GCP Guidelines is needed, to better reflect the current reality of independent clinical research.

By acknowledging the role and specificities of non-commercial sponsors, and by providing adapted guidance on standard research contracts, the international GCP codes would provide valuable guidance and support to non-commercial clinical research, whose relevance for global health is increasingly evident. This will only succeed if representatives of institutions involved in non-commercial clinical trials in the North and in the South (researchers, sponsors, administrators, legal experts etc.) are actively involved in the next, and increasingly urgent, GCP revision. It is of crucial importance that this review process is as inclusive, representative and transparent as possible.

## Abbreviations

CIOMS: Council for International Organizations of Medical Sciences
COHRED: Council in Health Research for Development
EU: European Union
GCP: Good Clinical Practices
ICH: International Conference for Harmonization
R&D: Research & Development
UK: United Kingdom
WHO: World Health Organization
